# Endovascular treatment versus best medical treatment for acute anterior cerebral artery occlusion: a single-center inverse probability of treatment-weighted cohort study

**DOI:** 10.3389/fneur.2026.1896249

**Published:** 2026-10-07

**Authors:** Xiaolin Wan, Baoming Lei, Kun Tang, Haifeng Liu, Yi Yang, Wenhua Liu, Zhangbao Guo

**Affiliations:** Department of Neurology, Wuhan No. 1 Hospital, Wuhan, Hubei, China

**Keywords:** acute ischemic stroke, anterior cerebral artery occlusion, best medical treatment, endovascular treatment, inverse probability of treatment weighting

## Abstract

**Introduction:**

Evidence regarding the benefit of endovascular treatment (EVT) for isolated acute anterior cerebral artery occlusion remains limited. We aimed to compare the efficacy and safety of EVT versus best medical treatment (BMT) in this population.

**Methods:**

We retrospectively screened consecutive patients with acute anterior cerebral artery occlusion between May 2020 and October 2025. The primary outcome was excellent functional outcome, defined as a 90-day modified Rankin Scale (mRS) score of 0–1. Secondary outcomes included 90-day mRS 0–2, mRS 0–3, mortality, symptomatic intracranial hemorrhage (sICH), and any intracranial hemorrhage (any ICH). Multivariable logistic regression and inverse probability of treatment weighting (IPTW) were used to adjust for baseline differences.

**Results:**

Among 69 included patients, 37 received BMT and 32 underwent EVT. Successful reperfusion was achieved in 27 of 32 patients treated with EVT (84.4%). The primary outcome of 90-day mRS 0–1 occurred in 17 of 37 patients (45.9%) in the BMT group and 13 of 32 patients (40.6%) in the EVT group. EVT was not significantly associated with 90-day mRS 0–1 after adjustment (adjusted odds ratio [OR], 1.74; 95% confidence interval [CI], 0.50–5.99). No significant differences were observed for 90-day mRS 0–2, mRS 0–3, mortality, sICH, or any ICH. IPTW-adjusted sensitivity analyses yielded consistent results.

**Conclusion:**

EVT showed high reperfusion success but was not associated with a statistically significant improvement in 90-day functional outcomes compared with BMT. Further multicenter studies are needed to clarify the potential role of EVT in this population.

## 1 Introduction

Medium-vessel occlusions (MeVO) account for approximately 25 to 40% of acute ischemic strokes (AIS) and represent a major therapeutic challenge (1, 2). Recent multicenter randomized clinical trials (RCTs) have provided inconsistent evidence regarding the efficacy of endovascular treatment (EVT) compared with best medical treatment (BMT) in patients with MeVO. Hu et al. (3) reported a potential benefit of EVT, trials conducted by Goyal et al. (4) and Psychogios et al. (5) did not demonstrate superiority of EVT over BMT. Among MeVO, evidence regarding the optimal treatment strategy for isolated anterior cerebral artery occlusion (ACAO) is particularly scarce.

Isolated ACAO accounts for only approximately 2% of all AIS (6–9). Patients with acute ACAO often present with clinically disabling symptoms, with motor deficits typically more prominent in the lower limb than in the upper limb (9). In addition, ACAO may cause a wide range of neuropsychological manifestations because of involvement of the frontal lobe and related structures, such as the corpus callosum and basal ganglia (6, 10). Depending on the occlusion site and vascular anatomical variations, ACAO can result in substantial long-term disability and reduced post-stroke quality of life (8).

For patients with acute ACAO and severe or disabling symptoms, intravenous thrombolysis remains a standard reperfusion therapy when administered within the recommended time window (11, 12). During the past decade, EVT has become the cornerstone of treatment for AIS caused by anterior or posterior circulation large-vessel occlusion (12, 13). However, the role of EVT in ACAO remains uncertain. Although EVT may achieve rapid reperfusion in patients with persistent occlusion and disabling deficits, the smaller caliber, distal location, and anatomical variability of ACA branches may increase technical difficulty and hemorrhagic risk (14, 15). Because patients with isolated ACAO have been underrepresented in EVT trials, treatment decisions are largely individualized in routine practice. Consequently, the efficacy, safety, and selection criteria for EVT in ACAO remain to be clarified.

To address this knowledge gap, we conducted a single-center cohort study to compare the efficacy and safety of EVT versus BMT in patients with acute ACAO admitted within 12 h of symptom onset.

## 2 Methods

### 2.1 Study design and population

This was a single-center retrospective cohort study of consecutive patients with acute ACAO admitted between May 2020 and October 2025. Patients were eligible for inclusion if they met the following criteria: (1) age ≥18 years; (2) acute ischemic stroke with ACAO confirmed by computed tomography angiography (CTA), magnetic resonance angiography (MRA), or digital subtraction angiography (DSA); (3) admission within 12 h from symptom onset or last known well; and (4) available 90-day follow-up. Patients were excluded if they had bilateral ACAO, concomitant large-vessel occlusion in other vascular territories, missing key baseline or imaging data, or loss to 3-month follow-up. The final cohort was divided into the BMT group and the EVT group according to the actual treatment strategy.

This study involves human participants and was approved by the institutional research ethics committees of the Wuhan No. 1 Hospital (No 2021–27). Written informed consent was obtained from all patients or their legally authorized representatives. This pooled analysis was conducted in accordance with the Declaration of Helsinki.

### 2.2 Treatment groups

Treatment allocation was neither randomized nor blinded. Treatment strategy was determined by the treating stroke neurologists and neuro-interventionalists according to patients’ status. BMT was defined as guideline-based medical management without endovascular treatment. It included antiplatelet therapy, anticoagulation when appropriate, lipid-lowering therapy, blood pressure and glucose management, and other standard stroke care. Intravenous thrombolysis was administered to eligible patients according to current guideline recommendations. EVT was performed at the discretion of the treating neurointerventional team. EVT techniques included stent retriever thrombectomy, contact aspiration, combined thrombectomy techniques, or rescue therapy when necessary. In patients undergoing EVT, procedural variables, including onset-to-puncture time, puncture-to-recanalization time, and final reperfusion status, were recorded. Reperfusion was assessed using the Medium-Vessel Occlusion–Expanded Thrombolysis in Cerebral Infarction (MeVO-eTICI) score (1), which ranges from 0 to 3, with higher scores indicating greater reperfusion. Successful reperfusion was defined as a MeVO-eTICI score of 2b or higher.

### 2.3 Data collection

Baseline demographic, clinical, imaging, procedural, and outcome data were retrospectively collected from the institutional stroke database and medical records. Baseline variables included age, sex, vascular risk factors, prior ischemic stroke, smoking status, baseline National Institutes of Health Stroke Scale score (16), systolic and diastolic blood pressure on admission, occlusion site, and use of intravenous thrombolysis. Vascular risk factors included hypertension, hyperlipidemia, diabetes mellitus, atrial fibrillation, and coronary heart disease. The occlusion site was categorized as A2 or A3 segment occlusion according to baseline vascular imaging.

### 2.4 Outcomes measurement

The primary outcome was excellent functional outcome at 90 days, defined as a modified Rankin Scale score of 0–1. Secondary outcomes included functional independence at 90 days, defined as mRS score 0–2; ambulatory outcome at 90 days, defined as mRS score 0–3. The 90-day mRS was assessed by trained investigators who were blinded to the treatment received. Safety outcomes included all-cause mortality at 90 days, symptomatic intracranial hemorrhage, and any intracranial hemorrhage at 48 h. Symptomatic intracranial hemorrhage was defined as any intracranial hemorrhage associated with neurological deterioration according to the Heidelberg Bleeding Classification (17). Any intracranial hemorrhage included all radiologically confirmed intracranial hemorrhage events regardless of clinical symptoms.

### 2.5 Statistical analysis

Continuous variables were presented as median with interquartile range and categorical variables as number with percentage. Baseline characteristics were compared between the BMT and EVT groups using the Mann–Whitney U test for continuous variables and the *χ*^2^ test or Fisher’s exact test for categorical variables, as appropriate. Given the limited sample size, we estimated the minimal detectable difference for the primary outcome based on the available sample sizes (37 patients in the BMT group and 32 in the EVT group), assuming a two-sided α of 0.05, 80% power, and the observed primary-outcome rate in the BMT group as the reference event rate.

Multivariable logistic regression was then used to estimate adjusted odds ratios and 95% confidence intervals for the association between treatment strategy and clinical outcomes. Given the limited sample size and the risk of model overfitting, covariates were selected *a priori* based on clinical relevance. For the primary and secondary efficacy outcomes, the adjusted model included age, baseline NIHSS score, occlusion site, and intravenous thrombolysis. For safety outcomes, including 90-day mortality, symptomatic intracranial hemorrhage and any intracranial hemorrhage, a more parsimonious model adjusting only for baseline NIHSS score was used because of the limited number of events. An E-value analysis was performed for the primary outcome based on the multivariable-adjusted odds ratio to assess the potential impact of unmeasured confounding.

To further account for baseline imbalances between treatment groups, inverse probability of treatment weighting (IPTW) was performed as a sensitivity analysis. Propensity scores for receiving EVT were estimated using a logistic regression model including age, sex, baseline NIHSS score, occlusion site, and intravenous thrombolysis. Stabilized inverse probability of treatment weights were calculated for the average treatment effect. Covariate balance was assessed using absolute standardized mean differences (SMDs), with an absolute SMD < 0.10 considered indicative of adequate balance. Propensity-score distributions were visually inspected to assess common support. The distribution of stabilized weights was also examined to identify extreme weights. Stabilized weights ranged from 0.53 to 2.68 (mean, 0.99), and no extreme weights were identified; therefore, no weight trimming or truncation was applied.

All statistical analyses were performed using R software (version 4.5.0, R Foundation for Statistical Computing, Vienna, Austria). IPTW estimation, covariate balance diagnostics, and weighted outcome analyses were performed using the WeightIt (version 1.5.0), cobalt (version 4.6.1), and survey (version 4.4–8) packages, respectively. A two-sided *p* value <0.05 was considered statistically significant. No adjustment for multiple comparisons was performed; therefore, analyses of secondary outcomes and safety outcomes should be interpreted as exploratory.

## 3 Results

### 3.1 Patient characteristics

Between May 2020 and October 2025, a total of 105 patients with acute ACAO admitted within 12 h from symptom onset or last known well were screened. After excluding patients with bilateral anterior cerebral artery occlusion, concomitant large-vessel occlusion in other vascular territories, missing key baseline data, or loss to 3-month follow-up, 69 patients were included in the final analysis. Among them, 37 patients received BMT and 32 patients underwent EVT (Figure 1). With the available sample size, assuming a two-sided α of 0.05 and 80% power, the study could detect an increase in the primary-outcome rate from 45.9% in the BMT group to approximately 78.0% in the EVT group, corresponding to an absolute difference of approximately 32.1%. Thus, the study was powered to detect only a large treatment effect.

**Figure 1:**
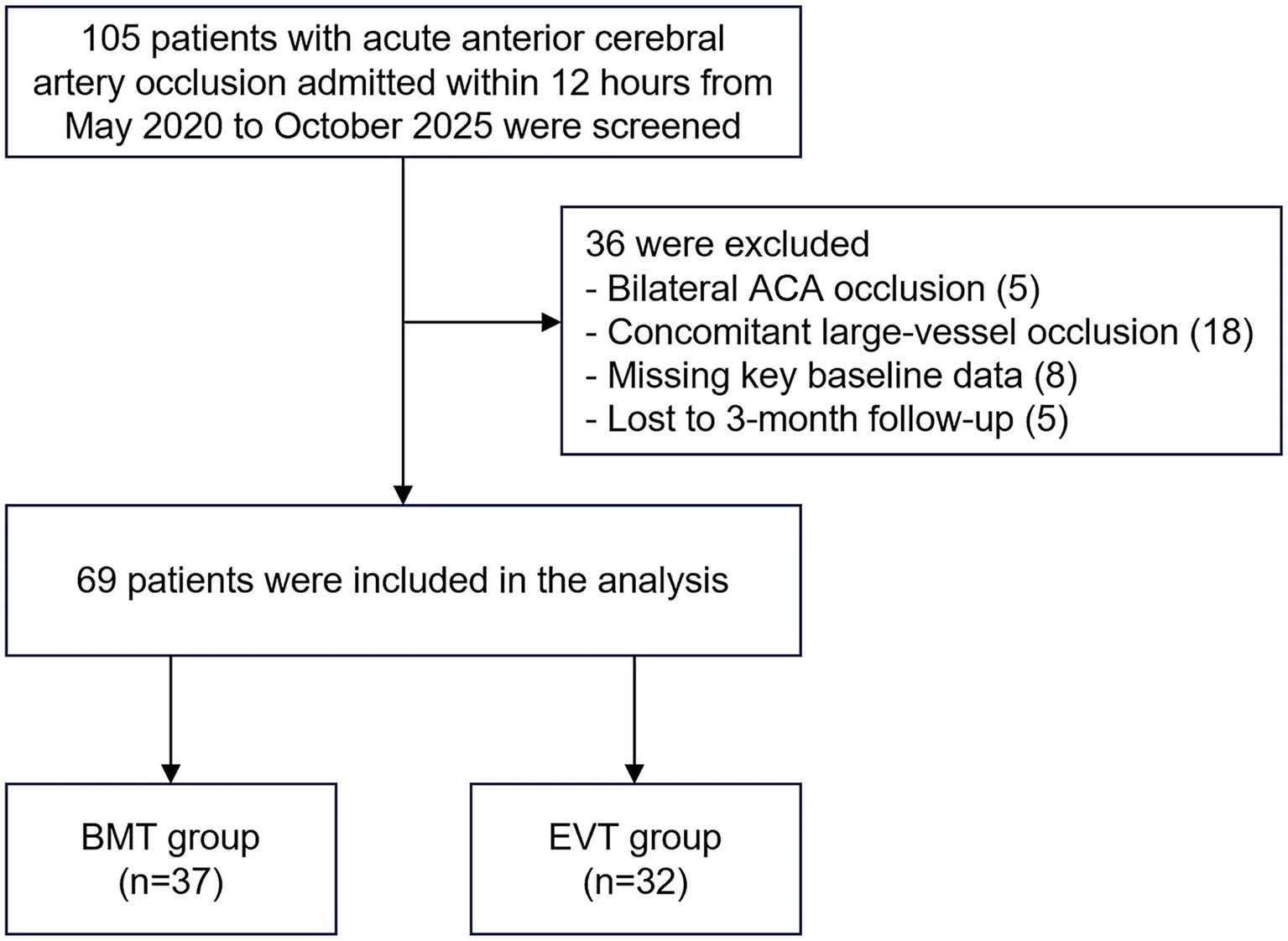
Flowchart of patient selection. ACA, anterior cerebral artery; BMT, best medical treatment; EVT, endovascular treatment.

Baseline characteristics according to treatment group are shown in Table 1. The median age was 66.0 (61.0–71.0) years, and 19 (27.5%) patients were female. The prevalence of vascular risk factors was generally comparable between the BMT and EVT groups. The use of intravenous thrombolysis was also similar between the two groups. Patients in the EVT group had a higher median NIHSS score (7.0 [5.5, 9.8]) than those in the BMT group (3.0 [2.0, 6.0]). A2 occlusion was the most common occlusion site in both groups. After IPTW, all covariates included in the propensity score model achieved adequate balance (absolute SMD < 0.10), including baseline NIHSS score, for which the SMD decreased from 0.524 to 0.046 (Supplementary Figure S1). Propensity-score distributions showed substantial overlap between the BMT and EVT groups (Supplementary Figure S2). The effective sample sizes after weighting were 34.0 and 27.2 in the BMT and EVT groups, respectively. Among patients treated with EVT, the median onset-to-puncture time was 336.5 (251.3, 532.5) minutes, and the median puncture-to-recanalization time was 101.5 (74.8, 147.0) minutes. Successful reperfusion was achieved in 27/32 (84.4%) patients.

**Table 1:** Baseline characteristics by treatment group before and after inverse probability of treatment weighting.

Characteristics	All patients (*n* = 69)	BMT (*n* = 37)	EVT (*n* = 32)	Unweighted *p*-value	SMD before IPTW	SMD after IPTW
Age, year, median (IQR)	66.0 [61.0, 71.0]	67.0 [61.0, 72.0]	66.0 [60.0, 69.3]	0.373	0.166	0.044
Sex, female, *n* (%)	19 (27.5)	10 (27.0)	9 (28.1)	1.000	0.025	0.048
Medical history, *n* (%)						
Hypertension	53 (76.8)	27 (73.0)	26 (81.2)	0.599	0.198	0.155
Hyperlipidemia	9 (13.0)	5 (13.5)	4 (12.5)	1.000	0.016	0.003
Diabetes mellitus	17 (24.6)	9 (24.3)	8 (25.0)	1.000	0.03	0.021
Atrial fibrillation	7 (10.1)	4 (10.8)	3 (9.4)	1.000	0.048	0.015
Coronary heart disease	7 (10.1)	3 (8.1)	4 (12.5)	0.839	0.145	0.056
Ischemic stroke	19 (27.5)	10 (27.0)	9 (28.1)	1.000	0.025	0.130
Smoking	34 (49.3)	19 (51.4)	15 (46.9)	0.897	0.09	0.033
Baseline NIHSS, median (IQR)	6.0 [2.0, 8.0]	3.0 [2.0, 6.0]	7.0 [5.5, 9.8]	<0.001	0.524	0.047
Systolic blood pressure, mmHg, median (IQR)	150.0 [134.0, 160.0]	150.0 [135.0, 160.0]	145.0 [132.3, 157.0]	0.466	0.086	0.095
Diastolic blood pressure, mmHg, median (IQR)	84.0 [78.0, 95.0]	83.00 [76.0, 95.0]	84.0 [79.5, 91.3]	0.647	0.14	0.208
Occlusion site, *n* (%)				0.232	0.371	0.002
A2	55 (79.7)	27 (73.0)	28 (87.5)			
A3	14 (20.3)	10 (27.0)	4 (12.5)			
Intravenous thrombolysis, *n* (%)	8 (11.6)	4 (10.8)	4 (12.5)	1.000	0.053	0.022
Onset to puncture time, min, median (IQR)	NA	NA	336.5 [251.3, 532.5]	NA		
Puncture to recanalization time, min, median (IQR)	NA	NA	101.5 [74.8, 147.0]	NA		
Successful reperfusion, n (%)	NA	NA	27 (84.4)	NA		

BMT, best medical treatment; EVT, endovascular treatment; IQR, interquartile range; IPTW, inverse probability of treatment weighting; NIHSS, National Institutes of Health Stroke Scale; SMD, standardized mean difference; NA, not available.

### 3.2 Primary and secondary outcomes

The primary outcome of 90-day mRS 0–1 occurred in 17/37 (45.9%) patients in the BMT group and 13/32 (40.6%) patients in the EVT group. In multivariable logistic regression, EVT was not significantly associated with 90-day mRS 0–1 compared with BMT (adjusted OR, 1.74; 95% CI, 0.50–5.99). The E-value for the adjusted primary-outcome estimate was 2.88; because the 95% CI included the null, the E-value for the confidence limit closest to the null was 1.00. For secondary outcomes, 90-day mRS 0–2 was observed in 23/37 (62.2%) patients in the BMT group and 20/32 (62.5%) patients in the EVT group. EVT was not significantly associated with 90-day mRS 0–2 after adjustment (adjusted OR, 2.53; 95% CI, 0.69–9.33). A 90-day mRS score of 0–3 was observed in 28/37 (75.7%) patients in the BMT group and 23/32 (71.9%) patients in the EVT group. The adjusted estimate was imprecise and did not reach statistical significance (adjusted OR, 7.72; 95% CI, 0.84–70.85). The distribution of 90-day mRS scores before and after IPTW is shown in Figure 2.

**Figure 2:**
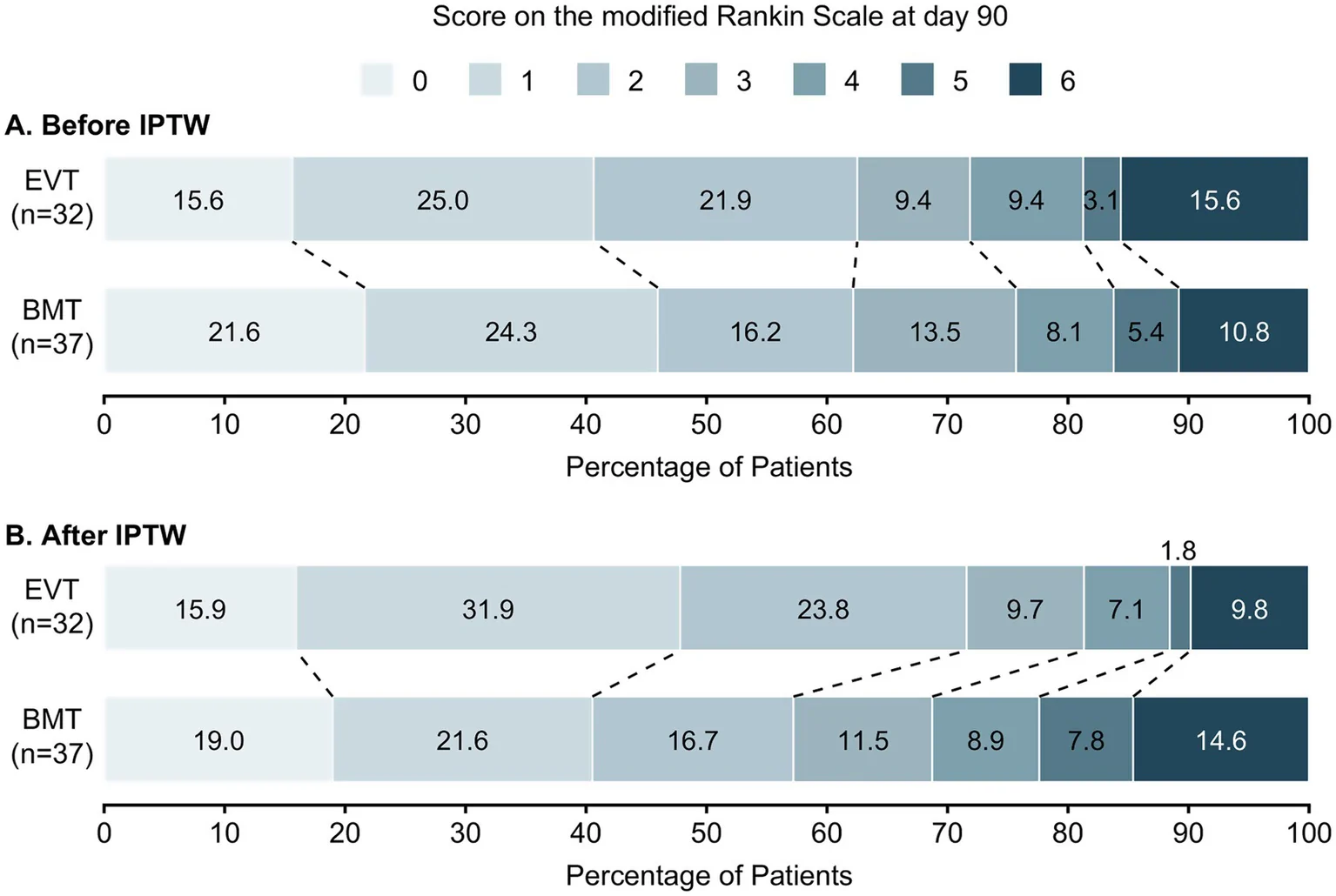
Distribution of modified Rankin Scale scores at 90 days compared according to treatment cohorts of endovascular treatment (EVT) and best medical treatment (BMT). **(A)** Before and **(B)** after inverse probability of treatment weighting. BMT, best medical treatment; EVT, endovascular treatment; IPTW, inverse probability of treatment weighting; mRS, modified rankin scale.

### 3.3 Safety outcome

At 90 days, mortality occurred in 4/37 (10.8%) patients in the BMT group and 5/32 (15.6%) patients in the EVT group. EVT was not significantly associated with 90-day mortality after adjustment (adjusted OR, 0.06; 95% CI, 0.00–2.54). Symptomatic intracranial hemorrhage occurred in 1 patient in the BMT group and 2 patients in the EVT group, with no significant difference after adjustment (adjusted OR, 1.38; 95% CI, 0.12–18.91). Any intracranial hemorrhage occurred in 1 patient in the BMT group and 5 patients in the EVT group; this numerical difference did not reach statistical significance after adjustment (adjusted OR, 6.24; 95% CI, 0.66–58.95).

### 3.4 Sensitivity analysis

In IPTW-adjusted analyses, EVT was not significantly associated with 90-day mRS 0–1 (IPTW-adjusted OR, 1.34; 95% CI, 0.48–3.78), 90-day mRS 0–2 (IPTW-adjusted OR, 1.88; 95% CI, 0.66–5.39), or 90-day mRS 0–3 (IPTW-adjusted OR, 1.98; 95% CI, 0.63–6.24). The IPTW-adjusted estimates for safety outcomes were also not statistically significant, including 90-day mortality, symptomatic intracranial hemorrhage, and any intracranial hemorrhage (Table 2).

**Table 2:** Clinical outcomes according to treatment group.

Outcomes	All patients	BMT	EVT	Crude OR (95% CI)	Adjusted OR (95% CI)	IPTW-adjusted OR (95% CI)
Primary outcome						
90-day mRS 0–1	30/69 (43.5%)	17/37 (45.9%)	13/32 (40.6%)	0.80 (0.31–2.10)	1.74 (0.50–5.99)	1.34 (0.48–3.78)
Secondary outcomes						
90-day mRS 0–2	43/69 (62.3%)	23/37 (62.2%)	20/32 (62.5%)	1.01 (0.38–2.69)	2.53 (0.69–9.33)	1.88 (0.66–5.39)
90-day mRS 0–3	51/69 (73.9%)	28/37 (75.7%)	23/32 (71.9%)	0.82 (0.28–2.41)	7.72 (0.84–70.85)	1.98 (0.63–6.24)
Safety outcomes						
90-day mortality	9/69 (13.0%)	4/37 (10.8%)	5/32 (15.6%)	1.53 (0.37–6.26)	0.06 (0.00–2.54)	0.64 (0.14–2.93)
sICH	3/69 (4.3%)	1/37 (2.7%)	2/32 (6.2%)	2.40 (0.21–27.78)	1.38 (0.12–18.91)	1.71 (0.13–22.59)
Any ICH	6/69 (8.7%)	1/37 (2.7%)	5/32 (15.6%)	6.67 (0.74–60.42)	6.24 (0.66–58.95)	6.61 (0.65–67.24)

BMT, best medical treatment; CI, confidence interval; EVT, endovascular treatment; ICH, intracranial hemorrhage; mRS, modified Rankin Scale; OR, odds ratio; sICH, symptomatic intracranial hemorrhage.

## 4 Discussion

In this single-center retrospective cohort study of patients with acute ACAO admitted within 12 h, EVT achieved a high rate of angiographic success in our cohort, with successful reperfusion in 84.4%. EVT was not significantly associated with a higher likelihood of excellent functional outcome (mRS 0–1) at 90 days compared with BMT. Similar findings were observed for 90-day mRS 0–2 and mRS 0–3. Safety outcomes, including 90-day mortality, sICH, and any ICH, also did not differ significantly between the two treatment groups.

Our findings are particularly relevant in the context of the current research focus on MeVO stroke (18, 19). In The Endovascular Treatment to Improve Outcomes for Medium Vessel Occlusions (ESCAPE-MeVO), EVT did not improve the primary endpoint of 90-day mRS 0–1 (41.6% vs. 43.1%) and was associated with higher mortality and more sICH (4). Likewise, in The Endovascular Therapy plus Best Medical Treatment versus BMT Alone for Medium Vessel Occlusion Stroke (DISTAL), EVT did not improve the primary ordinal mRS distribution, and secondary excellent outcome rates were similar between groups (5). By contrast, Hu et al. (3) reported that, among patients with MeVO and moderate-to-severe deficits, EVT increased the likelihood of functional independence at 90 days, with a numerically higher sICH rate but no corresponding increase in mortality. Thus, our neutral results align more closely with ESCAPE-MeVO and DISTAL than with the positive signal observed by Hu et al. Importantly, however, direct cross-trial comparisons should be made cautiously because the primary endpoints, eligibility criteria, baseline severity, and imaging selection strategies differed substantially across trials (20–22). Several differences may partly explain the apparently discrepant findings. Unlike the randomized MeVO trials, our study was restricted to isolated A2/A3 occlusions and treatment was selected in routine clinical practice, with patients undergoing EVT having substantially greater neurological severity at baseline, thereby increasing the potential for confounding by indication. In addition, differences in patient eligibility, occlusion territories, imaging selection, and procedural strategies across studies may have contributed to the heterogeneous treatment-effect estimates.

The angiographic findings in our cohort are particularly noteworthy. Our successful reperfusion rate of 84.4% was numerically higher than those reported in the major MeVO RCTs, including 75.1% in ESCAPE-MeVO (4) and 71.7% in DISTAL (5), and was also high relative to large observational MeVO registries (23). This reinforces a central lesson emerging from the literature: technical success is necessary, but not sufficient, for functional benefit in MeVO stroke. Meyer et al. (15) reported comparable 90-day functional outcomes between thrombectomy and medical management despite technical feasibility, and Salim et al. (14) similarly found high procedural success (91%) without a significant improvement in functional independence or mortality. Similarly, a retrospective international multicenter study by Candice Sabben et al. (24) reported that 49% of patients with ACAO treated with BMT alone still experienced poor functional outcomes. Our cohort extends this line of evidence to a single-center, real-world ACAO population, further highlighting the need to clarify the potential role of EVT in this specific MeVO subtype.

The apparent mismatch between reperfusion and outcome in our cohort may have several explanations. First, EVT patients had more severe baseline deficits, raising the likelihood of confounding by indication despite multivariable adjustment and IPTW. Second, the sample was small, with wide confidence intervals. Third, dichotomized mRS may undercapture ACA-specific disability, including abulia, executive dysfunction, behavioral change, gait impairment, and leg-predominant weakness (6, 8, 9). Fourth, ACA thrombectomy is technically distinct from more proximal EVT: the vessel is smaller, more distal, anatomically variable, and perforator-rich, so angiographic reopening may not fully reflect downstream tissue salvage or microvascular reperfusion (25, 26).

Safety deserves equally careful discussion. In our cohort, symptomatic intracranial hemorrhage was uncommon and not significantly different between groups, but any intracranial hemorrhage was numerically more frequent after EVT. This signal is directionally consistent with several MeVO studies. ESCAPE-MeVO showed more sICH and higher mortality with EVT (4); DISTAL showed numerically higher sICH without a major mortality difference (5); Hu et al. also reported a higher sICH rate with EVT but similar 90-day mortality (3); and by contrast, some observational registries did not find a statistically significant safety penalty (14). Mechanistically, ACA thrombectomy may be vulnerable to hemorrhagic complications because of smaller vessel caliber, distal catheter navigation, more fragile vessel walls, perforator-rich anatomy, and the possibility that multiple device manipulations can increase endothelial injury or reperfusion-related bleeding (27, 28). These risks may be particularly relevant for more distal occlusions, tortuous vascular anatomy, or patients with unfavorable infarct-core or collateral profiles (29–31). Therefore, the absence of a statistically significant safety difference in our study should not be overinterpreted as proof of procedural equivalence.

The clinical implication of our study is not that EVT should be abandoned for isolated ACAO, but that it should not be considered routine for all such patients. A more defensible approach is selective EVT for patients with proximal A2 occlusion, clearly disabling deficits (including severe leg weakness, profound abulia, aphasia, or marked functional dependence), small infarct core, favorable perfusion mismatch or collateral profile, and anatomy amenable to safe distal access in an experienced center. Conversely, patients with mild or nondisabling symptoms, distal and technically hazardous occlusions, large established infarction, or uncertain tissue viability may be better served by optimized medical therapy alone. Future studies should move beyond simple “EVT versus BMT” comparisons and instead identify which ACAO phenotype is most likely to benefit from reperfusion.

### 4.1 Limitations

Several limitations should be acknowledged. First, this was a single-center retrospective study with a limited sample size, which reduced statistical power and resulted in wide confidence intervals for several adjusted estimates. The study was powered to detect only a large absolute difference (~32%) in the primary outcome; therefore, smaller clinically meaningful treatment effects cannot be excluded. Second, treatment allocation was not randomized, and patients selected for EVT had more severe baseline neurological deficits, suggesting potential confounding by indication. Although adjusted regression and IPTW were used, unmeasured confounding may remain. Third, the number of safety events was small, limiting the reliability of adjusted estimates for hemorrhagic outcomes and mortality. Fourth, detailed procedural characteristics and periprocedural complications were not systematically available. Finally, detailed imaging variables, such as infarct core volume, perfusion mismatch, collateral status, thrombus burden, and occlusion length, were not systematically available.

## 5 Conclusion

In this single-center cohort of patients with isolated ACAO, EVT was associated with high angiographic reperfusion but was not significantly associated with better 90-day excellent functional outcome than BMT. The direction and magnitude of the estimates were imprecise, and the findings are best viewed as exploratory in light of the small sample size and likely confounding by indication.

## Data Availability

The raw data supporting the conclusions of this article will be made available by the authors, without undue reservation.
