# Supplementary material for: Endovascular treatment versus best medical treatment for acute anterior cerebral artery occlusion: a single-center inverse probability of treatment-weighted cohort study
Source: Front Neurol. 2026 Oct 7;17:1896249. doi: 10.3389/fneur.2026.1896249 (PMC13683190; doi:10.3389/fneur.2026.1896249)
Supplement: Supplementary file 1 [file Supplementary_file_1.docx]

eFigure 1 Covariate balance before and after inverse probability of treatment weighting.

Absolute standardized mean differences are shown for baseline covariates before and after inverse probability of treatment weighting. The dashed vertical line represents the prespecified balance threshold of 0.10. Covariate balance was substantially improved after weighting.

Abbreviations: ASPECTS, Alberta Stroke Program Early CT Score; BMT, best medical treatment; EVT, endovascular treatment; IPTW, inverse probability of treatment weighting; NIHSS, National Institutes of Health Stroke Scale; IVT, intravenous thrombolysis; SMD, standardized mean difference.

eFigure 2. Distribution of propensity scores according to treatment group.

Density plots show the distribution of estimated propensity scores for patients receiving best medical treatment (BMT) and endovascular treatment (EVT). Substantial overlap between the two treatment groups was observed, supporting adequate common support for the inverse probability of treatment weighting analysis.
